# Safety and Efficacy of Misoprostol versus Oxytocin for the Prevention of Postpartum Hemorrhage

**DOI:** 10.1155/2014/713879

**Published:** 2014-03-05

**Authors:** Minoo Rajaei, Samieh Karimi, Zohreh Shahboodaghi, Hamidreza Mahboobi, Tahereh Khorgoei, Farzam Rajaei

**Affiliations:** ^1^Hormozgan Fertility and Infertility Research Center, Hormozgan University of Medical Sciences (HUMS), Bandar Abbas, Iran; ^2^Infectious and Tropical Diseases Research Center, Hormozgan University of Medical Sciences (HUMS), Bandar Abbas 7914964153, Iran; ^3^Department of Psychology, Payame Noor University (PNU), Tehran, Iran; ^4^Shiraz University of Medical Sciences (SUMS), Shiraz, Iran

## Abstract

Postpartum hemorrhage (PPH) is the commonest cause of maternal death worldwide. Studies suggest that the use of misoprostol may be beneficial in clinical settings where oxytocin is unavailable. The aim of this study was to compare the safety and efficacy of oxytocin and misoprostol when used in the prevention of PPH. In a double-blind randomized controlled trial, 400 pregnant women who had a vaginal delivery were assigned into two groups: to receive either 20 IU of oxytocin in 1000 mL Ringer's solution and two placebo tablets or 400 mcg oral misoprostol (as two tablets) and 2 mL normal saline in 1000 mL Ringer's solution. The quantity of blood loss was higher in the oxytocin group in comparison to the misoprostol group. There was no significant difference in the decrease in hematocrit and hemoglobin between the two groups. Although there was no significant difference in the need for transfusions between the two groups, the patients in the oxytocin group had greater need for additional oxytocin. Results from this study indicate that it may be considered as an alternative for oxytocin in low resource clinical settings. This study is registered with ClinicalTrials.gov NCT01863706.

## 1. Background

Postpartum hemorrhage (PPH) is a life-threatening obstetric emergency that occurs after caesarean section (CS) or normal vaginal delivery (NVD). It may be defined as ≥500 mL hemorrhage after vaginal or ≥1000 mL hemorrhage after CS delivery [1–3]. PPH is one of the most common obstetric maternal complications and is among the three most common etiologies of maternal death worldwide [4]. Its incidence is increasing and it affects 1–5% of all deliveries [5, 6]. Atony is the main cause of PPH and is responsible for about 80% of PPHs [7]. Therefore, uterotonic agents are administered. Oxytocin infusion, single dose of methylergometrine, and then carboprost tromethamine are used in 15-to-20-minute intervals in atony. Misoprostol, which is a prostaglandin E1 analog, is an inexpensive drug and can be absorbed by the following routes of administration: vaginal, rectal, or oral (sublingual or buccal absorption) [8, 9]. Gastrointestinal symptoms (nausea, vomiting, and diarrhea) and fever are the most common adverse effects of misoprostol, which often are mild and self-limited [10–12].

Several studies have shown that misoprostol is more effective than oxytocin and methylergometrine in the treatment of PPH [13, 14]. Although misoprostol can be used as first-line therapy in the treatment of PPH where oxytocin is not available [15], other studies have not confirmed that misoprostol is more effective than oxytocin in the prevention of PPH.

The aim of this study was therefore to compare the efficacy and safety of oral misoprostol and intravenous oxytocin in the prevention of PPH.

## 2. Method

This double-blind randomized controlled trial was undertaken at Shariati Hospital, the only obstetrics and gynecology educational hospital in Bandar Abbas. Bandar Abbas is the capital and the main city of Hormozgan province, located in southern Iran.

The study was approved by the Research Committee of Hormozgan University of Medical Sciences (HUMS) and is registered with ClinicalTrials.gov (ClinicalTrials.gov Identifier: NCT01863706). Written informed consent was obtained from all the patients.

The required sample size was calculated to be at least 175 patients in each study group (based on weighting pads) considering the standard deviation of 100 gr for hemorrhage in each group for detecting a 30 gr difference between two groups (*α* = 0.05; *β* = 0.20).

The inclusion criteria were women with singleton pregnancy with cephalic presentation who had NVD spontaneously or by induction.

The exclusion criteria were placenta previa (based on ultrasound sonography in the third trimester), placental abruption, coagulation problems, previous CS, macrosomia (defined as estimated fetal weight above 4000 gr based on ultrasound sonography), polyhydramnios (defined as amniotic fluid index more than 24 cm), and uncontrolled asthma.

Women were randomly assigned to one of two groups with a 1 : 1 allocation using simple randomization with computerized random numbers: 20 IU oxytocin in 1000 mL Ringer's solution at a rate of 600 mL/hr plus placebo misoprostol tablet (group 1: routine management for prevention of PPH) or 400 *μ*g oral misoprostol as the alternative active management of the third stage of labour plus placebo oxytocin in 1000 mL Ringer's solution at a rate of 600 mL/hr for prevention of PPH (Group 2).

For blinding the study identical appearing solutions and tablets corresponding to the two pharmacological groups were prepared by the pharmacy and kept in the fridge until required. A blood sample was obtained before delivery and 24 hours after delivery. In addition, the quantity of blood loss was calculated by weighing the pads utilised after NVD. To calculate this, the pads which were used after NVD were weighed before usage and after usage (after absorbance of blood). By this method we obtained the blood loss amount in grams. Also, vital signs and drug-adverse effects were recorded using a standard checklist.

The primary outcome was hemorrhage (quantified blood loss) within 1 hour of delivery; the secondary outcomes were hematocrit (HCT) and hemoglobin (Hb) 24 hours after delivery, hemodynamic instability within 24 hours after delivery, and drug-adverse effects, including fever (≥38°C), chills (reported by the patient), diarrhea, nausea, vomiting, and hypotension. Any additional requirements for oxytocin or blood transfusion were recorded. Data was analysed using SPSS 16 software and chi-square and independent samples *t*-test analysis. Results are presented as mean and standard deviation and the data are normally distributed.

## 3. Results

Four hundred pregnant women were included in the study. The mean age of the study participants was 25.86 ± 5.79 years. Two hundred patients were included in each group. There were not significant differences in the gestational age, birth weight and past perineal tear in the groups.

Table 1 compares the primary and secondary outcome between the two groups. There was no significant difference in amount of hemorrhage, Hb, HCT, Hb decrease, or HCT decrease between the two groups. The rate of need for additional oxytocin was higher in group 1, but the rate of adverse effects was higher in group 2.

## 4. Discussion

In this study we compared the safety and efficacy of misoprostol and oxytocin in the prevention of PPH. Our analysis showed that there was no statistically significant difference in the baseline characteristics in the two groups.

The primary outcome in our study was amount of hemorrhage. Our study showed a significant decrease in hemorrhage when misoprostol was used to prevent PPH compared to oxytocin treatment. This finding is compatible with the finding of the study by Lokugamage et al. [14] who reported the superiority of misoprostol over Syntometrine in managing PPH. They used intramuscular Syntometrine (Sandoz Pharmaceuticals) (ampoule = 5 iu oxytocin and 500 mcg ergometrine maleate) plus Syntocinon (Sandoz Pharmaceuticals) (10 iu oxytocin diluted in 500 mL normal saline) intravenous infusion versus 800 mcg misoprostol rectally. However, Lokugamage et al. used rectal misoprostol rather than oral used in the current study. Further, the current study examined the use of misoprostol for the prevention of PPH, but Lokugamage et al. used misoprostol for *treatment* of PPH. Other studies have shown better efficacy for misoprostol in comparison to methylergometrine in the prevention of PPH [13].

The differences in the results of our study compared with other similar studies which have used oxytocin intravenously or intramuscularly may be explained by the rate of oxytocin which is given to the patients. We used 20 IU oxytocin in 1000 mL Ringer's solution at a rate of 600 mL/hr.

Widmar et al. reported different results. They reported no significant difference between 600 mcg misoprostol sublingually and a placebo in patients who were under routine treatment for uterine contraction [16]. Their results are different from our study because they studied the efficacy of misoprostol in oxytocin-resistant PPH. Similar findings are reported in the studies by Winikoff et al. [15] and Blum et al. [17]. They found no significant difference between using sublingual 800 mcg misoprostol and 40 IU intravenous oxytocin for PPH [15, 17]. Both studies were conducted for the treatment of PPH and the outcome was stopping bleeding more than 300 mL within 20 minutes after delivery.

In our study there was no significant difference between Hb and HCT or Hb and HCT decrease between the two groups 24 hours after treatment. Other studies have reported lower HCT decrease by using misoprostol in comparison to ergometrine.

In our study there was additional use of oxytocin in group 1. In a study by Haque et al., 94% of the patients had no need for additional oxytocin and only 6% of the patients had moderate hemorrhage and additional oxytocin was added. Also 2% of the patients in the oxytocin group in this study needed additional oxytocin [18].

The fever rate was higher in the misoprostol group in our study, but there was no significant difference between the two groups in chills and gastrointestinal symptoms. This finding was confirmed by Blum et al. [15], Haque et al. [16], and Baskett et al. [17].

In our study, 0.5 and 2% of the patients in the oxytocin and the misoprostol groups needed blood transfusions, which was no statistically significant difference. Baskett et al. and Haque et al. reported no cases of transfusion need in their studies [18, 19]. In this study patients received blood transfusion based on Hb or HCT decrease. Therefore no patient received transfusion before measuring the Hb and HCT decrease (within the first hour of the treatment). Therefore blood transfusion had no effect on these outcomes.

Multicentre studies with higher sample sizes and meta-analysis studies are still needed to obtain the optimal treatment strategies in the prevention of PPH.

## 5. Conclusion

Our study suggests that the use of misoprostol is more effective for decreasing the amount of blood loss, thereby avoiding a PPH, and is associated with mild and self-limiting side effects. Misoprostol is cost effective and easily administered and therefore may be considered for use in low resource areas when oxytocin is unavailable.

## Figures and Tables

**Table 1:** Comparison of study outcomes in the two groups.

Outcomes	Group 1 (*n* = 200)	Group 2 (*n* = 200)	*P* value
Amount of hemorrhage (gr)	182.4 ± 101.3	157.0 ± 84.9	0.007
Hb (mg/dL)	10.9 ± 1.5	10.7 ± 1.4	0.243
HCT (%)	32.2 ± 4.2	32.7 ± 3.6	0.200
Hb decrease (mg/dL)	0.8 ± 1.1	0.7 ± 0.9	0.363
HCT decrease (%)	2.2 ± 3.3	2.1 ± 2.7	0.629
Additional oxytocin	21 (10.5%)	9 (7.5%)	0.018
Any adverse effects	7 (3.5%)	30 (15%)	0.001
Transfusion	4 (2%)	1 (0.5%)	0.184
Hypotension*	1 (0.5%)	0 (0%)	0.5
Fever	4 (2%)	29 (14.5%)	*P* < 0.001
Chills	2 (1%)	2 (1%)	0.688

*Defined as systolic BP ≤ 90 mmHg or diastolic BP ≤ 60 mmHg.
